# Recurrent Cervical Cancer Treated Successfully with Single-Agent PARP-Inhibitor, Olaparib

**DOI:** 10.1155/2022/6579715

**Published:** 2022-10-25

**Authors:** Maya Gross, Ryan J. Spencer

**Affiliations:** ^1^Department of Obstetrics and Gynecology, University of Wisconsin-Madison School of Medicine and Public Health, USA; ^2^Division of Gynecologic Oncology, University of Wisconsin-Madison School of Medicine and Public Health, USA

## Abstract

Recurrent cervical cancer has a grim prognosis with 5-year survival <5%. Current treatment options are limited; standards of care such as palliative chemotherapy and surgical resection often provide a small survival advantage. To date, only one targeted agent has FDA approval for the treatment of recurrent cervical cancer. We present the case of a novel application of olaparib, a poly (adenosine diphosphate-ribose) polymerase (PARP) inhibitor, as single-agent therapy for recurrent metastatic clear cell cervical cancer in a patient with a somatic BRCA2 mutation. The patient had excellent response to therapy with stable disease without evidence of progression until 14 months of therapy, at which time she was switched to an alternative regimen.

## 1. Introduction

Recurrent cervical cancer of any histologic type poses a grim prognosis. Women diagnosed with recurrent disease have a 5-year survival of <5% [1]. Decisions regarding treatment of recurrent disease are based on recurrence site and primary treatment course. While central recurrences are sometimes amenable to definitive surgical management with pelvic exenteration, treatment options for distant recurrences remain limited and overall survival is typically measured in months [1–3]. Palliative chemotherapy, traditionally delivered with cisplatin-based regimens, has been one of the only available options for recurrent or metastatic cervical cancer not amenable to resection [4]. For patients with tumors positive for Programmed Death-Ligand 1 (PD-L1), Pembrolizumab has been approved by the FDA for treatment of patients with recurrent or metastatic cervical cancer following disease progression on or after chemotherapy [5], while clinical trials have evaluated treatment with alternative targeted therapies; to date, none are FDA approved for this indication.

Classically associated with in utero diethylstilbestrol (DES) exposure, clear cell adenocarcinoma of the cervix remains a rare subtype of cervical cancer. Treatment regimens are similar to other adenocarcinomas of the cervix, as response to treatment and prognosis between subtypes is comparable [3].

We describe a clinical case of a patient with recurrent, metastatic, clear cell cervical adenocarcinoma who was treated with single-agent olaparib, a PARP-inhibitor, with robust and durable response to therapy.

## 2. Case Presentation

The patient is a 48-year-old G1P1 patient who presented to an academic gynecologic oncology practice as a referral for abnormal uterine bleeding and was ultimately diagnosed with stage IIB clear cell adenocarcinoma of the cervix based on a 7 × 7 × 2 cm invasive carcinoma with involvement of the upper 1/3 of the vagina and parametrial invasion. Imaging including CT and PET/MRI was without evidence of lymphadenopathy or distant spread. Due to her diagnosis of stage IIB cervical cancer, she was referred to radiation oncology for counseling on concurrent chemoradiation. Tumor genomics via Next Generation Sequencing were performed at time of initial referral demonstrating BRCA2 p.(Y1739^∗^) c.5217_5223delTTTAAGT mutation, which is a nonsense mutation changing tyrosine to a stop codon within coding exon 10. This variant has been identified as a germline variant; the patient declined genetic counseling and germline genetic testing [6]. She underwent primary treatment with 45Gy pelvic intensity-modulated radiotherapy and 25Gy high dose radiation cervical brachytherapy with concurrent weekly cisplatin, delivered over a ten-week period. Initial treatment course was complicated by development of a saddle pulmonary embolus with right heart strain and NSTEMI for which she was placed on therapeutic anticoagulation. Posttreatment follow-up exam and MRI demonstrated complete resolution of the cervical soft tissue mass without MR evidence of locoregional residual disease, and no metastatic disease within the pelvis.

She then attended surveillance visits, with no evidence of disease, until presenting to the emergency department with a second saddle pulmonary embolism after self-discontinuation of DOAC one year prior. Imaging performed at that time demonstrated numerous new solid and cystic metastatic lesions in the abdomen and pelvis with lesions along the liver capsule and peritoneum. Ultrasound-guided biopsy demonstrated carcinoma consistent with metastasis from the patient's known primary cancer. Progression-free survival time to first recurrence was 18 months.

Discussion regarding options for treatment of recurrent cervical clear cell carcinoma included traditional systemic chemotherapy regimens as well as consideration of previous tumor genomics result that demonstrated a somatic BRCA2 mutation. Upon discussion with the patient regarding results of molecular testing, along with recommendations from our institution's precision medicine molecular tumor board, we decided to initiate therapy with twice daily oral olaparib. The medication was approved by her insurance with the somatic BRCA2 mutation data and documented recommendation from the institutional molecular tumor board. Repeat CT scan prior to initiation of PARP-inhibitor demonstrated continued enlargement of numerous masses within the abdomen and pelvis consistent with worsening disease.

**Figure 1:**
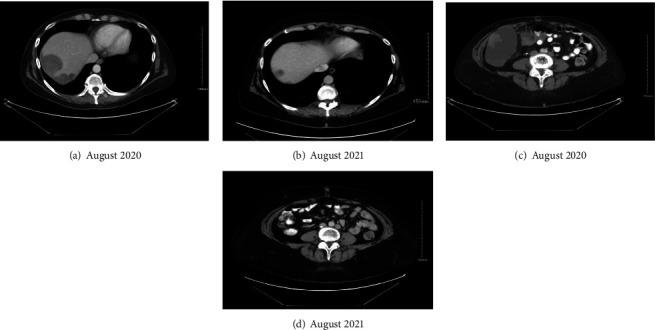
Interval decrease in disease burden over one year period of olaparib administration, including hepatic dome lesion from 58x42 mm (a) to 24x18 mm (b); lower right paracolic gutter lesion from 108x66 mm (c) to complete response (d).

After 3 months of treatment with single agent olaparib, repeat CTAP demonstrated interval decrease in tumor burden. Six- and 9-month surveillance visit imaging demonstrated continued interval decreases in tumor size and burden. Her dose was decreased at 9 months of treatment due to decreased GFR according to FDA package insert. At her 12-month surveillance visit, exam, and imaging was consistent with stable disease without evidence of interval progression (Figure 1). Response of specific target lesions from initiation of therapy to this 12-month time point were: hepatic dome from 58x42 mm to 24x18 mm; gall bladder fossa from 38x34 mm to complete response; upper right paracolic gutter 73x73 mm to 23x22 mm; lower right paracolic gutter from 108x66 mm to complete response; lower liver lobe from 41x32 mm to 32x21 mm; and right paraaortic lymph node conglomerate from 47x28 mm to 17x8 mm.

The patient continued on olaparib for 14 months, at which time she developed symptoms concerning for progression which was confirmed on imaging. She was counseled on options and was transitioned to systemic chemotherapy with cisplatin, paclitaxel, and bevacizumab.

## 3. Discussion

Recurrent cervical cancer of any histologic type is associated with a 5-year survival of <5% [1]. Treatment options for distant recurrences remain limited and overall survival is typically measured in months [1–3]. Palliative chemotherapy, traditionally delivered with cisplatin-based regimens, has been one of the only available options for recurrent or metastatic cervical cancer not amenable to resection [4].

Carboplatin-paclitaxel has become the standard of care for patients with high risk for nonresponse to cisplatin [1, 2]. The addition of bevacizumab to platinum-based chemotherapy doublets in GOG240 offered a novel therapy, improving overall survival to 17 months from time of randomization vs. 13.3 months in the non-bevacizumab control group. Notably, roughly 20% of patients enrolled in GOG 240 had cervical adenocarcinoma, nearly 70% had recurrent disease, and patients identified as high-risk for nonresponse to cisplatin derived greater survival benefit from the addition of bevacizumab than did low-risk patients [1, 2].

Investigations into alternative options have revealed promising targets, with several studies attempting to capitalize on mutations in cervical cancer tumors. The Keynote-028 and Keynote-158 trials demonstrated durable, well-tolerated response to pembrolizumab, an immune checkpoint inhibitor which binds to PD-L1 in patients with PD-L1 positive tumors [7–9]. Pembrolizumab has since been approved by the FDA for treatment of patients with recurrent or metastatic cervical cancer following disease progression on or after chemotherapy whose tumors express PD-L1 [5]. Two separate studies have evaluated an alternative PD-L1 inhibitor, Nivolumab and have revealed discordant results [ 10, 11]. CheckMate358 demonstrated a durable, well-tolerated response with an overall response rate of 26%, while a phase II GOG trial (NRG-GY002) demonstrated a poor response rate, with only 4% of participants experiencing any response using RECIST 1.1 criteria. Although up to 77% of tumor specimens had quantifiable PD-L1 expression; response rates were not correlated to degree of PD-L1 expression in either study [10, 11].

Aside from the above-mentioned therapies, few options exist for treatment of distant metastatic cervical cancer. Several other antiangiogenic agents have been studied for treatment of cervical cancer, including sunitinib, pazopanib, lapatinib, and cediranib. While initial studies of these molecules have been promising, with some demonstrating improved progression-free survival, to date no other antiangiogenic molecule has been shown to meaningfully impact outcomes via improved overall survival [1, 9].

Olaparib is a PARP-inhibitor, a class of drugs which targets the DNA repair mechanism present in tumor cells, blocking the ability to fix DNA single-strand breaks leading to a greater degree of DNA double-strand breaks. In patients with BRCA mutations who thus have a homologous recombination repair deficiency, these double-strand breaks lead to a greater degree of cell death which is more pronounced in the molecularly deranged malignant cells. PARP-inhibitors have been utilized in multiple cancers and patients with germline and/or somatic BRCA mutations as a method to remove the tumor's back-up method of DNA repair. The utilization of PARP-inhibitors for treatment of recurrent or metastatic cervical cancer has theoretical plausibility. Several studies have demonstrated increased rate of BRCA mutations in cervical cancer tumors [12, 13]. An investigative group which performed theranostic evaluation of cervical cancer specimens demonstrated that 21% of tumors had BRCA2 mutations and 10% had BRCA1 mutations [13]. Another study demonstrated in vitro efficacy of olaparib when used as a single-agent as well as when used as a sensitizing agent with concurrent cisplatin administration [14]. Documentation of frequent mutations in BRCA1 and BRCA2 as well as in vitro efficacy against cervical cancer cells grants biological plausibility to the targeted treatment of cervical cancer with PARP-inhibitors, as well as for routine tumor genomic assessment of cervical cancer specimens.

Two trials have evaluated treatment of recurrent or metastatic cervical cancer with the PARP-inhibitor veliparib, in combination with other therapies already in use for treatment of cervical cancer [9, 12, 15, 16]. In NCT #01281852, which evaluated veliparib in combination with paclitaxel and cisplatin, an objective response rate was achieved in 34% of patients with measurable disease; median PFS was 6.2 months and overall survival was 14.5 months [15]. Results from NSC #737664, which evaluated use of veliparib in combination with topotecan and GCSF demonstrated poor response with a partial response rate of 7%, median PFS of 2 months as well as significant toxicity [16]. One case report was recently published documenting complete response to combination therapy with olaparib and bevacizumab in a patient with advanced cervical cancer [17]. Additional trials are ongoing with PARP-inhibitors rucaparib and niraparib in combination with bevacizumab and concurrent radiation therapy, respectively [18, 19].

To the authors' knowledge, no trial has been initiated evaluating use of olaparib in the treatment of recurrent or metastatic cervical cancer. Additionally, existing trials and case reports have not investigated single-agent PARP-inhibitor therapy. This case report documents durable response to single-agent PARP-inhibitor therapy with olaparib in a patient with recurrent cervical cancer with distant metastases. The patient had significant partial response to therapy with some lesions responding completely and others with a more modest response; recurrence developed at 14 months after initiation of treatment.

### 3.1. Conclusions

Recurrent cervical cancer with distant metastases has limited treatment options with an extremely poor prognosis, with overall survival for these patients estimated at 1-1.5 years [2, 9]. Despite the addition of novel therapies such as bevacizumab and pembrolizumab, patients suffering from a distant recurrence, especially those whose tumors do not demonstrate PD-L1 activity have gained a survival benefit of a few months when compared to traditional treatment options.

Biological plausibility exists for treating recurrent cervical cancers with gene-specific or targeted therapies. As documented above, multiple studies are currently underway evaluating targeted therapies alone or in combination with current standard of care treatments. Future studies should investigate the utility of olaparib in the treatment of recurrent cervical cancer, both alone and as a combination therapy. As the patient in this case report has a diagnosis of clear cell adenocarcinoma of the cervix, further studies should evaluate efficacy of PARP-inhibitors in patients with squamous cell or other subtypes of cervical cancer. Further evaluation of cost effectiveness of routine vs. selective analysis of cervical cancer tumors via tumor genomics with Next Generation Sequencing may be beneficial. Evaluation of cervical cancer tumors with tumor genomics may offer insights for predicting potential response to targeted agents, as review of the literature demonstrates that many tumors may exhibit potentially targetable mutations.

In this patient with a distant recurrence of clear cell cervical adenocarcinoma with a somatic BRCA2 mutation, treatment with single-agent olaparib resulted in a durable response of 14 months with limited toxicity. While several other studies have demonstrated biological plausibility of treatment of recurrent cervical cancer with PARP-inhibitors, to the author's knowledge this is the first case report documenting in vivo success with single-agent olaparib and response to olaparib in this patient has outlasted currently available data from trials evaluating other PARP-inhibitors in the treatment of cervical cancer.

## Data Availability

The data supporting this case report comes from previously reported studies and datasets, which have been cited. The processed data are available from the corresponding author upon request.
